# Health-related quality of life of individuals sharing a household with persons with dementia

**DOI:** 10.1007/s11136-021-03065-z

**Published:** 2021-12-17

**Authors:** Judith Dams, Thomas Grochtdreis, Hans-Helmut König

**Affiliations:** Department of Health Economics and Health Services Research, University Medical Center Hamburg-Eppendorf, Martinistraße 52, 20246 Hamburg, Germany

**Keywords:** HrQoL, Dementia, Social environment, SF-12, Households

## Abstract

**Introduction:**

Previous research has found a negative effect of dementia on the health-related quality of life (HrQoL) of persons with dementia (PWD) and their primary informal caregivers. However, the impact of dementia on HrQoL of other individuals sharing a household with PWD has not been investigated to date. The current study therefore aimed to determine differences in the HrQoL between those sharing a household with PWD and those not living with PWD. In addition, factors related to the HrQoL of those sharing a household with PWD were evaluated.

**Methods:**

The analyses were based on data from the German Socio-Economic Panel, using the SF-12 to measure HrQoL. Mixed-effects models were calculated to compare the HrQoL of those sharing a household with PWD and persons not living with PWD, as well as to determine factors related to the HrQoL of those sharing a household with PWD. Bootstrapping was used where residuals were not normally distributed.

**Results:**

Mixed-effect models showed a significantly lower HrQoL among those sharing a household with PWD, compared to those not living with PWD. Number of diseases, number of persons in the household, marital status and educational level were significantly related to HrQoL among those sharing a household with PWD.

**Discussion:**

The HrQoL of those sharing a household with PWD was reduced compared to persons not living with PWD. Further, those living with PWD in small households, or those with multi-morbidities had a lower HrQoL. Further research focusing on HrQoL in the social environment of PWD is needed.

## Plain English summary

Little is known about health-related quality of life (HrQoL) in the social environment of persons with dementia (PWD), with the exception of caregivers. This article examined the HrQoL of individuals sharing a household with PWD, and compares it to those not living with PWD, using a representative survey of German households. Results revealed a reduced HrQoL among those sharing a household with PWD. The health status of the individual, and the total number of persons in the household, had a major impact on HrQoL. No difference was found in the HrQoL of those sharing a household with PWD, who were also the primary informal caregivers, and other persons in the household. Further research focusing on HrQoL in the social environment of PWD is needed.

## Introduction

Dementia is the most common mental illness in old age, with approximately 1.5 million people affected in Germany [1]. The prevalence is estimated to be 1.5% of persons aged 65 (*n* = 64,300) and 40% of persons aged 90 (*n* = 281,700) [1]. Due to demographic changes, the overall number of PWD is expected to increase in the coming decades, so that the number of PWD will double by the year 2050 to approximately 3.1 million [1].

In the early stages of the disease, dementia patients usually live at home, and care is often provided informally by relatives and friends [2–4]. In particular, care is provided by persons who are married or partnered (60%), who have a higher level of education (37%) and who have no children (68%). The majority of informal caregivers (50%) are in full-time employment, whilst 43% are unemployed [5]. In more than 60% of these cases, informal care is provided by women [6]. In the later stages of the disease, cognitive and physical impairments worsen, so that half of patients require 35 h of care, or more, per week [7]. At this stage, professional care is needed [4, 8]. Furthermore, with increasing cognitive and physical impairments, HrQoL of PWD deteriorates [9]. In particular, HrQoL decreases when residential care is unavoidable and patients have to leave their familiar living environment [9].

Informal care demands are associated with physical strain and mental distress for primary informal caregivers [10]. As a significant amount of time is spent providing informal care, approximately 43% of the primary informal caregivers are not employed, particularly if care is being provided to individuals in later stages of dementia [11]. Due to negative consequences on everyday life, primary informal caregivers often suffer from mental illnesses, such as depression, anxiety or stress [12, 13], as well as a reduced HrQoL [10]. A recently published systematic review showed heterogeneous results among international studies on the HrQoL of primary informal caregivers for PWD [10]. Overall, socio-demographic characteristics, caregiver–patient relationship, dementia characteristics (duration of dementia, age of onset, functional impairment, comorbidities), demands of caring, caregiver health, caregiver emotional well-being, support received, caregiver independence, caregiver self-efficacy, and worrying about the future were found in 41 studies to be associated with HrQoL of persons primarily providing informal care. Only one study analyzed the HrQoL of primary informal caregivers in Germany [14].

Caring for PWD not only affects the HrQoL of those living with PWD, who are also the primary informal caregiver, but also that of other household members [12, 13]. Especially in the case of dementia, household members are faced with an additional burden with respect to care and supporting PWD in their everyday life. Furthermore, the person who acts as the primary informal caregiver often have to balance work, caregiving and their personal interests [15]. It seems reasonable, that similar to the primary informal caregiver, individuals who live with a PWD may also experience be physically strain and/or mental stress, which in turn would influence their HrQoL negatively. Although the severity of dementia varies from person to person, the relationship between PWD and family members and friends are likely to change over time due to changed communication (e.g. PWD forget names and words) and altered behavior (some PWD become more impatient or aggressive) [16]. These difficulties can place an additional burden on family members and friends. Even though existing household surveys [e.g. the German Socio-Economic Panel (SOEP)] provide data to assess the impact of dementia on household members, the HrQoL of those sharing a household with PWD, who are not primary informal caregivers has not been addressed in the literature. It is important to address this gap in order to identify the possible health impacts for those sharing a household with PWD.

Therefore, the aim of this study was to build on existing literature on the HrQoL of persons who are the primary caregivers for PWD, by including all household members of PWD. Based on the SOEP, the HrQoL of individuals sharing a household with PWD was compared with the HrQoL of persons not living with PWD (*research question 1*). In addition, determinants of HrQoL among those sharing a household with PWD were assessed (*research question 2*).

## Methods

### Data

The analyses were based on SOEP data, provided by the German Institute for Economic Research (DIW Berlin), which has interviewed more than 20,000 household members of a representative sample of all German households annually since 1984. Data for 35 waves are available up until 2019. Data is collected using different questionnaires for individuals, households or specific subgroups. Based on the subgroups, and comprising frequently used variables, SOEP core data sets are prepared by the DIW Berlin. The SOEP core data sets related to household and personal questionnaires, including sociodemographic data of adult participants, as well as data on health, were used for the current analyses.

Core datasets of household questionnaires were used to capture the relationship between household members. In comparison to other representative surveys, which usually only interview one person in a household, the SOEP survey seeks to interview all household members. According to the methods report of the DIW Berlin, only household members indicating important reasons for non-attendance were not included in the study. Thus, questionnaires from all household members within a panel wave were available for 95% of all households [17].

Core datasets related to personal questionnaires with health-related data included information on chronic diseases and HrQoL. HrQoL for adult participants was surveyed every even year since 2002. In order to select individuals sharing a household with PWD, PWD were determined by self-disclosure, based on the question “Has a doctor ever diagnosed you to have dementia?” which was surveyed every odd year since 2009. Data on individuals sharing a household with PWD were limited tdco four waves before the dementia diagnosis was first reported, in order to reflect the undiagnosed, initial phase of dementia. Data of all waves after a dementia diagnosis were considered. PWD living alone were excluded. While the SOEP was designed as a household survey, and persons from one household were followed up even if they moved away from the primary household, our analysis only included data on individuals sharing a household with PWD while they were still living with the PWD. Data on persons not living with PWD were not restricted by household characteristics. At the project start, data for the year 2018 (wave 34) were not released by the DIW Berlin and thus not included in the current analyses. Thus, longitudinal data for the even waves between 2006 and 2016 (waves 22 to 32) were used. Within the SOEP, minors were not interviewed on their HrQoL, thus the current analyses were based on data from adults only.

### Health-related quality of life

Since 2006, the SOEP questionnaire includes a modified version of the Short Form-12 (SF-12) questionnaire to measure HrQoL every second year [18–20]. The SF-12 comprises 12 questions from the Short Form-36, each being answered on a 3 to 5-step ordinal scale. Derived scores were *Z*-transformed using means and standard deviations of a normative sample of the German population [20, 21]. Subsequently, the Physical Component Summary (PCS) score was calculated by summing up the *Z*-scores for questions addressing physical health, impaired physical role function, physical pain and general health. Similarly, the *Z*-scores for questions addressing mental health, limited emotional role function and social functioning were combined to the Mental Component Summary (MCS) score. The PCS and MCS represent the physical and mental HrQoL on a scale between 0 (worst HrQoL) and 100 (best HrQoL), respectively.

In addition to the MCS and PCS, SF-6D index scores were derived from questions on physical health, limited physical/emotional role function, physical pain, vitality, mental health, and social functioning using preference-based value sets for the British population [22]. Thus, for each of the 7.500 possible health states, an index value between 0 (death) and 1 (full health) was calculated [22].

### Sociodemographic and clinical characteristics

Information on age, gender (female and male), marital status (married, single, widowed, divorced, and separated), educational level, number of diseases, informal caregiving (errands outside the home, running the household, preparing meals, minor and major care), and the total number of persons in the household was considered. As the German education system differs from international education systems, educational level was measured by the years until the highest graduation was achieved. Total number of persons in the household corresponded to the number of completed personal questionnaires per household per wave.

The duration of dementia was estimated based on the years since first diagnosis. Furthermore, the number of additional diseases was calculated. For this purpose, participants were asked within the SOEP whether a doctor has ever diagnosed one or more diseases, such as sleep disorder, diabetes, asthma, cardiac disease, cancer, stroke, migraine, high blood pressure, depression, joint diseases, and chronic back pain.

The persons in need of care within a household was identified using the core datasets of household questionnaires and through the use of the personal identification number. A need for informal care for dementia was assumed where the person in need of care was also the person identified to be diagnosed with dementia. The person who was the primary informal caregiver in the household was identified based on a corresponding question in the household questionnaire.

### Statistical analyses

In order to minimize the number of missing values in sociodemographic characteristics and HrQoL, missing values on age, gender and marital status were replaced by values from other waves. Missing values of the HrQoL scales (PCS, MCS and SF-6D index) varied between 24% for households with PWD and 31% for households without PWD. It is recommended that missing values are imputed in studies with missing rates above 5% [23]. Multivariate imputation by chained equations (MICE) was therefore performed in this study, with *m* = 20 imputations to replace missing values [24].

Descriptive statistics of sociodemographic characteristics of individuals sharing a household with PWD and persons not living with PWD were calculated.

Mixed-effects models were calculated to compare the HrQoL of individuals sharing a household with PWD, with the HrQoL of persons not living with PWD, longitudinally (*research question 1*). HrQoL was used as dependent variable, whilst each respective group was included using a dummy-coded independent variable. Adjustments were made for age, gender, marital status, educational level, number of diseases, total number of persons in the household, being or not being the primary informal caregiver and survey year to consider group differences in sociodemographic and clinical characteristics. Subgroup analyses compared the HrQoL of those sharing a household with PWD, who were also the primary informal caregiver, with the HrQoL of persons not living with PWD. Furthermore, differences in HrQoL among those sharing a household with PWD, who were not primary informal caregivers, and persons not living with PWD were calculated. As residuals were normally distributed, no bootstrapping approach was applied.

Likewise, mixed-effects models were used to determine factors related to the HrQoL of those sharing a household with PWD (*research question 2*). Again, HrQoL was chosen as dependent variable. Age, gender, marital status, educational level, total number of persons in the household, being or not being the primary informal caregiver, and number of diseases were integrated into the model as independent variables. As residuals were not normally distributed, a bootstrapping approach with 1000 resamples was chosen.

All analyses were performed using R 3.5.1. The package “mice” was used for multiple imputation, which was supplemented by the package “miceadds” to calculate Spearman correlations. All applied statistics were two-sided. As Spearman correlations were used in the explorative analysis, no corrections for multiple testing were applied by calculating correlation coefficients. The level of significance in regression analyses was set at *α* = 0.017 (0.05/3) to correct for multiple significance tests and to avoid a type I error [25].

## Results

The current study included *n* = 213 individuals who share a household with PWD, of whom *n* = 60 (28%) were primary informal caregivers and *n* = 87 (*n* = 41%) were female. The mean age of individuals sharing a household with PWD was 57 years, whereas the mean age of persons not living with PWD (*n* = 66,218) was 47 years. Approximately 60% of those sharing a household with PWD were married, whereas 58% of persons not living with PWD were married and 28% were single. Thus, those sharing a household with PWD were older, more likely to be male, more likely to be widowed, had a higher number of diseases and lived with a lower number of persons within the household, compared with persons not living with PWD. Neither groups, however, differed with regard to their educational level. Among those sharing a household with PWD, the mean PCS score was 43.4, the mean MCS score was 46.4, and the mean SF-6D index scores was 0.66 in the year dementia was diagnosed. The mean PCS, MCS and SF-6D index scores of persons not living with PWD were 51.0, 49.8 and 0.74, respectively. The sociodemographic characteristics of the sample are shown in Tables 1 and 2.

**Table 1:** Sociodemographic and clinical characteristics of those sharing a household with persons with dementia and persons not living with a person with dementia

Sociodemographic and clinical characteristics	Individuals sharing a household with persons with dementia (*n* = 213)	Persons not living with persons with dementia (*n* = 66,218)	*p*-value
Measurement points: *n*			
4 years before diagnosis	146		
2 years before diagnosis	173		
Year when dementia was diagnosed	193		
2 years after diagnosis	98		
4 years after diagnosis	50		
6 years after diagnosis	28		
Age: mean (SD)	57 (24)	47 (17)	**
Female gender: *n* (%)	87 (41)	33,816 (51)	**
Marital status: *n* (%)			**
Married (ref.)	114 (59)	38,392 (58)	
Single	54 (28)	18,148 (28)	
Widowed	14 (7)	2905 (4)	
Divorced	6 (3)	4869 (7)	
Separated	5 (3)	1904 (3)	
Educational level: *n* (%)			
No school-leaving qualification	25 (13)	5801 (9)	
Certificate after grade 9 (ref.)	85 (44)	16,897 (26)	
Certificate after grade 10	41 (21)	16,883 (25)	
Higher education entrance qualification (grade 11)	5 (3)	3343 (5)	
Higher education entrance qualification (grade 12/13)	26 (13)	11,955 (18)	
Other	11 (6)	11,339 (17)	
Number of diseases: mean (SD)	1.9 (1.9)	0.1 (0.6)	**
Number of persons in the household: mean (SD)	2.3 (1.2)	3.9 (2.6)	**
Health-related quality of life: mean (SD)			
PCS	43.4 (10.6)	51.0 (9.8)	**
MCS	46.4 (11.9)	49.8 (11.0)	**
SF-6D index	0.66 (0.14)	0.74 (0.14)	**

Sociodemographic and clinical characteristics are presented for the year when dementia was diagnosed; *p*-value: comparison of categorical characteristics of those sharing a household with persons with dementia and persons not living with persons with dementia were analyzed using Pearson's *χ*^2^ test; comparison of mean age, number of diseases, number of persons in the household and health-related quality of life was analyzed using Student's *t*-test

*PCS* Physical Component Summary, *MCS* Mental Component Summary, *SF-6D*: Short-Form 6-Dimensions, *ref*. reference group

*p ≤ 0.007, **p ≤ 0.001

**Table 2:** Sociodemographic and clinical characteristics of individuals sharing a household with persons with dementia being the primarily informal caregiver and other person in the household

Sociodemographic and clinical characteristics	Individuals sharing a household with persons with dementia being the primarily informal caregiver (*n* = 60)	Other household members of persons with dementia (*n* = 153)	*p*-value
Measurement points: *n*			
4 years before diagnosis	47	99	
2 years before diagnosis	57	116	
Year when dementia was diagnosed	60	133	
2 years after diagnosis	28	70	
4 years after diagnosis	8	42	
6 years after diagnosis	3	25	
Age: mean (SD)	69 (14)	52 (26)	**
Female gender: *n* (%)	35 (58)	52 (39)	
Marital status: *n* (%)			
Married (ref.)	37 (62)	77 (58)	
Single	9 (15)	45 (34)	
Widowed	8 (13)	6 (5)	
Divorced	5 (8)	1 (0)	
Separated	1 (2)	4 (3)	
Educational level: *n* (%)			
No school-leaving qualification	2 (3)	23 (17)	
Certificate after grade 9 (ref.)	26 (44)	59 (44)	
Certificate after grade 10	13 (22)	28 (21)	
Higher education entrance qualification (grade 11)	2 (3)	3 (2)	
Higher education entrance qualification (grade 12/13)	12 (20)	14 (11)	
Other	5 (8)	6 (5)	
Number of diseases: mean (SD)	2.4 (1.8)	1.7 (2.0)	
Number of persons in the household: mean (SD)	2.3 (0.8)	2.4 (1.2)	
Health-related quality of life: mean (SD)			
PCS	42.6 (9.5)	43.8 (11.0)	
MCS	47.4 (10.4)	46.0 (12.5)	
SF-6D index	0.65 (0.12)	0.66 (0.15)	

Sociodemographic and clinical characteristics are presented for the year when dementia was diagnosed; *p*-value: comparison of categorical characteristics of individuals sharing a household with persons with dementia being the primarily informal caregiver and other persons in the household were analyzed using Pearson's *χ*^2^ test; comparison of mean age, number of diseases, number of persons in the household and health-related quality of life was analyzed using Student's *t*-test

*PCS* Physical Component Summary, *MCS* Mental Component Summary, *SF-6D* Short-Form 6-Dimensions, *ref*. reference group

*p ≤ 0.007, **p ≤ 0.001

In the mixed-effects models, those sharing a household with PWD had a significantly lower HrQoL compared with persons not living with PWD (Table 3). Among those sharing a household with PWD, a higher number of diseases was significantly associated with a difference in mean PCS, MCS, and SF-6D index scores of − 0.90 (*p* = 0.003), − 0.86 (*p* = 0.013), and − 0.01 (*p* < 0.001), respectively (Table 4). In addition, those sharing a household with PWD, who were also separated (*n* = 5) had lower MCS scores (− 11.46; *p* < 0.001) and lower SF-6D index scores (− 0.09; *p* = 0.005) compared to their married counterparts (*n* = 114). Individuals sharing a household with PWD, who graduated after 11 years in school (*n* = 5) had a significantly higher PCS score than those who’d not graduated (+ 6.92; *p* = 0.005; *n* = 25). PCS scores of those sharing a household with PWD, who lived in smaller households were significantly lower (− 5.99; *p* = 0.009), compared with those in larger households. Marital status, gender, age, and informal caregiving was not found to have a significant influence on the HrQoL of those sharing a household with PWD.

**Table 3:** Comparison of health-related quality of life of those sharing a household with persons with dementia compared with persons not living with persons with dementia using mixed-effects models

Sociodemographic characteristics	PCS			MCS			SF-6D index		
	Coefficient	Lower CI	Upper CI	Coefficient	Lower CI	Upper CI	Coefficient	Lower CI	Upper CI
Intercept	− 313.27***	− 341.42	− 285.12	− 274.67***	− 312.81	− 236.54	− 8.12***	− 8.60	− 7.64
Individuals sharing a household with PWD (ref. persons not living with PWD)	− 1.24*	− 2.21	− 0.28	− 2.56***	− 4.79	− 2.33	− 0.06***	− 0.07	− 0.05
Years since diagnosis	0.19***	0.17	0.20	0.16***	0.14	0.18	0.00***	0.00	0.00
Age	− 0.23***	− 0.24	− 0.23	0.09***	0.08	0.09	0.00***	0.00	0.00
Female gender (ref. male gender)	− 0.93***	− 1.05	− 0.82	− 1.91***	− 2.06	− 1.76	− 0.03***	− 0.03	− 0.03
Marital status (ref. married)									
Single	− 0.33***	− 0.49	− 0.17	− 0.28	− 0.51	− 0.05	− 0.01***	− 0.01	− 0.01
Widowed	− 0.35*	− 0.64	− 0.07	− 1.66***	− 1.98	− 1.34	− 0.03***	− 0.03	− 0.02
Divorced	− 0.27**	− 0.46	− 0.09	− 1.77***	− 2.04	− 1.49	− 0.02***	− 0.02	− 0.01
Separated	0.77***	0.47	1.06	− 3.61***	− 4.00	− 3.23	− 0.03***	− 0.04	− 0.02
Educational level (ref. no graduation)									
Certificate after grade 9	0.11	− 0.13	0.35	0.42**	0.12	0.72	0.01***	0.01	0.01
Certificate after grade 10	1.86***	1.64	2.07	0.92***	0.63	1.20	0.03***	0.02	0.03
Higher education entrance qualification (grade 11)	3.00***	2.70	3.30	0.93***	0.51	1.35	0.04***	0.03	0.04
Higher education entrance qualification (grade 12/13)	3.88***Ta	3.63	4.12	1.19***	0.89	1.49	0.05***	0.04	0.05
Other	0.49***	0.26	0.73	0.46**	0.13	0.78	0.01***	0.01	0.02
Number of persons in the household	− 0.04***	− 0.06	− 0.02	− 0.07***	− 0.09	− 0.04	0.00***	0.00	0.00
Number of diseases	− 1.34***	− 1.37	− 1.29	− 0.60***	− 0.65	− 0.55	− 0.02***	− 0.02	− 0.02

*CI* 95% confidence interval, *PCS* Physical Component Summary, *MCS* Mental Component Summary, *SF-6D* Short-Form 6-Dimensions, *PWD* person with dementia

*p ≤ 0.017, **p ≤ 0.010, ***p ≤ 0.001

**Table 4:** Determinants of health-related quality of life of those sharing a household with persons with dementia

Sociodemographic characteristics	PCS			MCS			SF-6D index		
	Coefficient	Lower CI	Upper CI	Coefficient	Lower CI	Upper CI	Coefficient	Lower CI	Upper CI
Intercept	40.92***	30.90	50.93	56.09***	45.26	66.93	0.71***	0.58	0.83
Years since diagnosis	0.31	− 0.06	0.68	0.36	− 0.04	0.76	0.01**	0.00	0.01
Age	− 0.06	− 0.17	0.05	− 0.05	− 0.16	0.06	0.00	0.00	0.00
Female gender (ref. male gender)	1.43	− 1.05	3.91	− 2.12	− 4.84	0.59	− 0.01	− 0.04	0.02
Marital status (ref. married)									
Single	2.55	− 1.81	6.89	− 3.65	− 8.16	0.86	− 0.01	− 0.06	0.04
Widowed	− 0.47	− 3.68	2.74	− 2.51	− 6.25	1.23	− 0.04	− 0.08	0.00
Divorced	1.52	− 4.45	7.50	− 0.62	− 6.84	5.61	0.00	− 0.07	0.07
Separated	1.10	− 4.05	6.25	− 11.46***	− 17.30	− 5.62	− 0.09**	− 0.15	− 0.03
Educational level (ref. no graduation)									
Certificate after grade 9	2.56	− 4.65	9.78	1.21	− 4.11	6.54	0.02	− 0.03	0.08
Certificate after grade 10	3.08	− 1.85	8.02	1.86	− 3.89	7.61	0.06	0.00	0.11
Higher education entrance qualification (grade 11)	6.92**	2.14	11.70	5.01	− 4.40	14.42	0.04	− 0.07	0.15
Higher education entrance qualification (grade 12/13)	2.08	− 7.43	11.60	3.31	− 1.86	8.49	0.07	0.01	0.12
Other	0.68	− 0.72	2.07	1.12	− 5.75	8.00	0.02	− 0.06	0.10
Number of diseases	− 0.90**	− 1.49	− 0.30	− 0.86*	− 1.53	− 0.19	− 0.01***	− 0.02	0.00
Other household members (ref. persons primarily providing informal care)	1.14	− 1.27	3.54	− 1.76	− 4.43	0.91	− 0.02	− 0.05	0.01
Number of persons in the household	5.99**	1.51	10.46	− 1.19	− 2.71	0.33	− 0.01	− 0.03	0.00

Mixed-effects model with covariates of individuals sharing a household with persons with dementia

*CI* 95% confidence interval, *PCS* Physical Component Summary, *MCS* Mental Component Summary, *SF-6D* Short-Form 6-Dimensions

*p ≤ 0.017, **p ≤ 0.010, ***p ≤ 0.00

Subgroup analyses revealed significantly lower MCS and SF-6D index scores among those sharing a household with PWD, who were also primary informal caregivers, compared with persons not living with PWD, whilst the PCS scores did not significantly differ (Table 5). Furthermore, individuals sharing a household of PWD, who did not act as the primary informal caregiver, had significantly lower PCS, MCS and SF-6D index scores than persons not living with PWD (Table 6).

**Table 5:** Comparison of health-related quality of life individuals sharing a household with persons with dementia being the primarily informal caregiver compared with persons not living with a person with dementia using mixed-effects models

Sociodemographic characteristics	PCS			MCS			SF-6D index		
	Coefficient	Lower CI	Upper CI	Coefficient	Lower CI	Upper CI	Coefficient	Lower CI	Upper CI
Intercept	− 313.17***	− 340.26	− 286.08	− 275.08***	− 313.06	− 237.09	− 6.33***	− 6.82	− 5.84
Individuals sharing a household with PWD being the primarily informal caregiver (ref. persons not living with a PWD)	0.16	− 1.36	− 1.69	− 3.86***	− 5.72	− 2.04	− 0.03***	− 0.06	− 0.01
Years since diagnosis	0.19***	0.17	0.20	0.16***	0.14	0.18	0.00***	0.00	0.00
Age	− 0.23***	− 0.24	− 0.23	0.09***	0.08	0.09	0.00***	0.00	0.00
Female gender (ref. male gender)	− 0.94***	− 1.06	− 0.82	− 1.91***	− 2.06	− 1.76	− 0.03***	− 0.03	− 0.03
Marital status (ref. married)									
Single	− 0.33***	− 0.48	− 0.19	− 0.28	− 0.52	− 0.05	− 0.01***	− 0.01	− 0.01
Widowed	− 0.36*	− 0.63	− 0.08	− 1.66***	− 1.97	− 1.34	− 0.02***	− 0.02	− 0.02
Divorced	− 0.27**	− 0.45	− 0.08	− 1.76***	− 2.03	− 1.50	− 0.02***	− 0.02	− 0.01
Separated	0.77***	0.49	1.05	− 3.60***	− 3.99	− 3.22	− 0.03***	− 0.04	− 0.03
Educational level (ref. no graduation)									
Certificate after grade 9	0.11	− 0.13	0.35	0.42**	0.12	0.71	0.01**	0.00	0.01
Certificate after grade 10	1.86***	1.65	2.06	0.92***	0.63	1.20	0.02***	0.02	0.03
Higher education entrance qualification (grade 11)	3.00***	2.71	3.29	0.93***	0.51	1.35	0.03***	0.03	0.04
Higher education entrance qualification (grade 12/13)	3.88***	3.64	4.11	1.19***	0.89	1.49	0.04***	0.04	0.05
Other	0.49***	0.26	0.73	0.46**	0.14	0.78	0.01***	0.01	0.01
Number of persons in the household	− 0.04***	− 0.06	− 0.02	− 0.07***	− 0.09	− 0.04	0.00***	0.00	0.00
Number of diseases	− 1.33***	− 1.37	− 1.29	− 0.60***	− 0.65	− 0.55	− 0.02***	− 0.02	− 0.02

*CI* 95% confidence interval, *PCS* Physical Component Summary, *MCS* Mental Component Summary, *SF-6D* Short-Form 6-Dimensions, *PWD* person with dementia

*p ≤ 0.017, **p ≤ 0.010, ***p ≤ 0.001

**Table 6:** Comparison of health-related quality of life of individuals sharing a household with persons with dementia not being the primarily informal caregiver compared with persons not living with a person with dementia using mixed-effects models

Sociodemographic characteristics	PCS			MCS			SF-6D index		
	Coefficient	Lower CI	Upper CI	Coefficient	Lower CI	Upper CI	Coefficient	Lower CI	Upper CI
Intercept	− 314.06***	− 342.97	− 285.16	− 274.69***	− 312.88	− 236.50	− 8.13***	− 8.60	− 7.67
Individuals sharing a household with PWD not being the primarily informal caregiver (ref. persons not living with a PWD)	− 1.85*	− 2.94	− 0.76	− 2.81***	− 4.23	− 1.38	− 0.06***	− 0.07	− 0.05
Years since diagnosis	0.19***	0.17	0.20	0.16***	0.14	0.18	0.00***	0.00	0.00
Age	− 0.23***	− 0.24	− 0.23	0.09***	0.08	0.09	0.00***	0.00	0.00
Female gender (ref. male gender)	− 0.93***	− 1.04	− 0.82	− 1.92***	− 2.07	− 1.77	− 0.03***	− 0.03	− 0.03
Marital status (ref. married)									
Single	− 0.32***	− 0.46	− 0.18	− 0.28	− 0.52	− 0.05	− 0.01***	− 0.01	− 0.01
Widowed	− 0.36*	− 0.64	− 0.08	− 1.65***	− 1.97	− 1.33	− 0.03***	− 0.03	− 0.02
Divorced	− 0.27**	− 0.45	0.09	− 1.76***	− 2.04	− 1.49	− 0.02***	− 0.02	− 0.01
Separated	0.77***	0.48	1.06	− 3.60***	− 3.98	− 3.22	− 0.03***	− 0.04	− 0.02
Educational level (ref. no graduation)									
Certificate after grade 9	0.12	− 0.11	0.36	0.42**	0.13	0.72	0.01***	0.01	0.01
Certificate after grade 10	1.86***	1.65	2.07	0.92***	0.63	1.20	0.03***	0.02	0.03
Higher education entrance qualification (grade 11)	3.00***	2.71	3.27	0.93***	0.51	1.35	0.04***	0.03	0.04
Higher education entrance qualification (grade 12/13)	3.88***	3.65	4.10	1.19***	0.89	1.49	0.05***	0.04	0.05
Other	0.50**	0.28	0.73	0.46**	0.13	0.78	0.01***	0.01	0.02
Number of persons in the household	− 0.04***	− 0.06	− 0.02	− 0.07***	− 0.09	− 0.04	0.00***	0.00	0.00
Number of diseases	− 1.33***	− 1.37	− 1.29	− 0.60***	− 0.65	− 0.55	− 0.02***	− 0.02	− 0.02

*CI* 95% confidence interval, *PCS* Physical Component Summary, *MCS* Mental Component Summary, *SF-6D* Short-Form 6-Dimensions, *PWD* person with dementia

*p ≤ 0.017, **p ≤ 0.010, ***p ≤ 0.001

## Discussion

Based on data from a representative household survey, to our knowledge, this study was the first to show that all individuals who share a household with PWD had a reduced HrQoL, compared to persons not living with PWD. As comparable international studies on HrQoL in the home environment are not available, literature on HrQoL of primary informal caregivers will be discussed. The HrQoL of persons primarily providing informal care to PWD is known to be reduced compared with other persons in the household [10, 26]. In particular, primary informal caregivers of PWD had a higher burden of care and a lower HrQoL than primary informal caregivers of individuals not suffering from dementia [27]. A higher burden of care was associated with a lower HrQoL [28]. Behavioral problems and psychological symptoms in particular have been found to constitute a significant aspect of the burden of primary informal caregiving [29]. At the same time, taking on such a task is never without consequences for the social environment. In the current study, differences in HrQoL between those sharing a household with PWD, who act as the primary caregiver, and other persons in the household, were not observed. However, it is conceivable that, similar to persons who are the primary informal caregiver, behavioral problems and psychological symptoms of PWD may also act as a burden for other household members. In this context, living with PWD can be a challenge, irrespective of whether the individual is acting as the primary informal caregiver, as individual needs are often put on hold, which in turn, could have negative health consequences.

Factors that were negatively related to the HrQoL of those sharing a household with PWD in the current study were the number of diseases and the total number of persons in the household. This was expected, since multimorbidity compromises health and thereby HrQoL [10]. Furthermore, HrQoL was significantly lower in smaller households than in larger households. Most households consisted of one or two other adults in addition to PWD. There was a significant difference in the PCS score between households with 1–3 persons (mean PCS score of 38–39) and households with 4 or 5 persons (mean PCS score of 44–45). As the total number of persons in a household increased, the mean age of those persons decreased. In households with an individual in addition to the PWD, persons had a mean age of 78, whereas in households with 5 or more members, the mean age was 36. At the same time, in households with a member in addition to the PWD, 18% of the household members reported being single, whilst this was true for 48% of the persons in households with 5 or more members. Therefore, smaller households seemed more likely to consist of married or partnered persons, whereas larger households seemed more likely to be joint or multi-generation households. However, the differences in HrQoL that are associated with the number of persons in the households cannot be attributed to age and marital status alone. Drawing on findings from the literature, HrQoL of primary informal caregivers was found to be related to demographic factors, caregiver–patient relationship, dementia characteristics (duration of dementia, age of onset, functional impairment, comorbidities), caregiver demands, caregiver health, caregiver emotional well-being, support received, caregiver independence, caregiver self-efficacy, and concerns about the future [10]. Similar to persons who act as the primary informal caregiver to the PWD, the results of the current study determined that the health of those sharing a household with PWD was related to their HrQoL. However, other factors described in the literature, that may also be relevant to the HrQOL of other household members, were not surveyed in the SOEP. As relationships between household members are very different and the social structures in individual households vary, the way in which factors influence the HrQoL those living with PWD is not currently well understood. The association between these factors and HrQoL appears to be multifactorial, in that several factors may influence each other.

### Future research

The extent to which factors related to the HrQoL of persons acting as primary informal caregivers to PWD are linked factors related to the HrQoL of other household members should be investigated in future research. Such a relationship is certainly conceivable due to cohabitation. The results of the current study, for example, were unable to identify any difference in the HrQoL of those sharing a household with PWD, who also act as the primary informal caregiver, and other household members. Unfortunately, the participants of the SOEP were not asked about the activities they undertake as part of informal care, so the relationship between informal care and HrQoL could not investigated in further detail in the current study. Therefore, further research is needed to determine whether similar reasons as described in literature for primary informal caregivers would also be responsible for the lower HrQoL among those sharing a household with PWD. Secondary activities in informal care, structural changes within the social network, different roles within the household, childcare or financial changes may also be associated with differences in the HrQoL among those who share a household with PWD. Future research should therefore examine the reasons and mechanisms behind reduced HrQoL, so that interventions to reduce the burden of care for those living with PWD can be defined.

### Strength and limitations

The SOEP included data on the social environment of PWD. As other studies have only considered the HrQoL of the primary informal caregiver, the current study extends existing knowledge by assessing HrQoL in the living environment, without restricting household members to a specific subgroup. This has been made possible by the representative data of the SOEP for German households. PCS and MCS scores of those not living PWD (51.0 and 49.8) in the current study were similar to PCS and MCS scores (51.4. and 49.4) of a representative German population sample [30]. The assessment of HrQoL within the SOEP is therefore likely to be valid. Furthermore, the current study was based on advanced statistical methods. Missing values were handled by MICE to avoid a loss of statistical power. Additionally, the skewness of HrQoL data was taken into account using a bootstrapping approach with 1000 replicates.

Even though the SOEP was designed as household survey, the panel design may have led to some limitations. First, dementia characteristics were not measured with a clinical score. Rather, the severity of dementia was estimated according to the duration of dementia. Therefore, regression analysis was not adjusted for specific symptoms of dementia, which may have influenced the results. Furthermore, diagnosis of dementia was self-reported by the participants of the SOEP, which may have led to incorrect identification of PWD. Second, in the analyses comparing HrQoL between those sharing a household with PWD and those not living with PWD were taken into account by adjusting for differences in socio-demographic characteristics in the mixed-effect models. Other socio-demographic factors such as persons’ role within the household, support or income were not considered, although these factors may also be related to HrQoL. Third, the sample sizes of those living with PWD and in particular persons who act as primary informal caregivers were small. Furthermore, data was not representative for households with PWD, because PWD may be less able to participate in such a survey, compared with healthy individuals. In addition, minors were not included. Thus, generalizability of the results may be limited. Lastly, some of the results of this study can only be regarded as preliminary and further research is needed. As the group sizes of those sharing a household with PWD and also being separated (*n* = 5) or having graduated after 11 years at school (*n* = 5) were small, the statistical significance of these influencing factors of HrQoL may not be generalizable.

## Conclusions

The HrQoL of those sharing a household with PWD was reduced compared with persons not living with PWD. In particular, those living with PWD in small households, and multi-morbid individuals sharing a household with PWD, had a lower HrQoL. No difference in HrQoL was observed between those sharing a household with PWD, who act as the primary caregiver, and other persons in the household. Further research focusing on the HrQoL in the social environment of PWD is needed. In particular, the association between the HrQoL of persons who act as the primary caregiver and other persons sharing a household with PWD should be investigated.

## Data Availability

The data can be applied for via the website of the German Institute for Economic Research (DIW), Berlin. It is available free of charge for scientific, non-commercial use.

## Code availability

The R code is available upon request from the corresponding author.
